# Retrospective Real-Life Data, Efficacy and Safety of Vismodegib Treatment in Patients with Advanced and Multiple Basal Cell Carcinoma: 3-Year Experience from a Spanish Center

**DOI:** 10.3390/ijerph20105824

**Published:** 2023-05-15

**Authors:** Angela Ayén-Rodríguez, Laura Linares-González, Carlos Llamas-Segura, Francisco Manuel Almazán-Fernández, Ricardo Ruiz-Villaverde

**Affiliations:** Dermatology Department, Hospital Universitario San Cecilio, 18016 Granada, Spain

**Keywords:** nonmelanoma skin cancer, locally advanced basal cell carcinoma, hedgehog inhibitor, vismodegib

## Abstract

Background: Basal cell carcinoma (BCC) is the most common type of skin cancer and can represent a therapeutic challenge in patients with locally advanced disease. Vismodegib is a hedgehog pathway inhibitor approved by the FDA for use in this type of tumor. We present a case series to describe our experience with the use of vismodegib. Methods: A retrospective study that included patients treated with vismodegib at our dermatology unit was conducted. Monthly follow-up was performed, and we registered the clinical evolution and adverse reactions. Results: A total of six patients with locally advanced BCCs were included (50% males and 50% females), with a mean age of 78.5 years old. The treatment was administered over a mean of 5 months. A complete response was observed in four cases and partial response in two cases. No recurrence was detected, with a median follow-up duration after discontinuation of 18 months. Most patients (83%) had at least one adverse event, and two needed dose adjustment temporarily or permanently to continue. The main adverse effect was muscle spasms (66.7%). The main limitation of our study was the small sample, which was not representative of the general population. Conclusions: Vismodegib is a safe and effective treatment for locally advanced BCC, and its role in unresectable BCC seems to be an important option in these challenging cases.

## 1. Introduction

Basal cell carcinoma (BCC) is the most common type of skin cancer, with an unknown incidence and mortality due to the existence of numerous unreported cases [1,2]. GLOBOCAN estimated a million two hundred new cases of non-melanoma skin cancers (NMSCs), excluding BCC, in 2020 [3]. In addition, it has been estimated that nearly 85% of NMSCs are BCC, which, extrapolated to GLOBOCAN data, would result in an estimated 8 million new BCC cases [4]. On the other hand, BCC is the most common cancer in Caucasians.

Risk factors include cumulative ultraviolet light exposure, immunosuppression, a history of previous radiotherapy, and genetic disorders (e.g., nevoid basal cell carcinoma syndrome (NBCCS) or Gorlin’s syndrome) [5]. A central role in the development of BCC is played by the overactivation of the hedgehog (Hh) signaling pathway through inhibition of the transmembrane protein patched homolog 1 (PTCH1) or, less often, the activation of the transmembrane protein smoothened homolog (SMO) [6]. The Hh pathway is also an important regulator of patterning and growth during embryogenesis and this is normally inactivated in adults. Mutations that drive aberrant activation of the Hh pathway are present in most sporadic BCC, leading to uncontrolled cell proliferation and tumor growth.

In most cases, BCC has an indolent growth and, due to its low metastatic potential, is treated with surgery or local destruction methods [7]. Mohs micrographic surgery is the treatment of choice for BCC in cosmetically sensitive areas such as the face. Unfortunately, there is a subtype of advanced forms of BCC, locally advanced BCC (laBCC), that may cause a considerable disruption of organ functions, cosmetic deformation, and a negative impact on quality of life, representing 0.8% of BCC [8]. LaBCC is defined according to the National Comprehensive Cancer Network as “primary or recurrent disease that is not amenable to surgery or radiation therapy” [9], and its treatment represents a major therapeutic challenge. Non-surgical treatments such as imiquimod, topical 5-fluorouracil and photodynamic therapy have been essayed in these types of tumors, but reported results are mixed [10]. Palliative radiation may be an option to be considered in advanced disease for bleeding or large tumors, but this is limited by lesion location or the presence of genetic syndromes associated with increased skin cancer risk and involving a relative contraindication [11]. The introduction of Hh pathway inhibitors has led to a significant change in the management of laBCC. These drugs are nowadays the recommended systemic treatment for laBCC for which surgery or radiotherapy is inappropriate. Until now, the US Food and Drug Administration (FDA) and the European Medicines Agency (EMA) have approved two inhibitors of SMO for the treatment of laBCC that has recurred following surgery or radiotherapy or for those who are not eligible for surgery or radiotherapy, as well as metastatic BCC, where vismodegib was the first selective Hh inhibitor approved in 2012. Phase I and II trials demonstrated the clinical benefits of vismodegib. However, safety analyses showed frequent adverse events (AEs) associated with treatment (98%) [12]. The common AEs observed during treatment with vismodegib are class-related effects (common between vismodegib and sonidegib), and it can be difficult for patients to endure, which impacts treatment adherence. In addition, creatin phosphokinase (CK) levels, electrolytes level, renal function and liver function tests should be monitored [13].

The aim of this study is to describe our experience with the use of vismodegib for advanced and/or multiple BCCs in real-life practice between 2019 and 2022. This study involved a small sample size as a consequence of the criteria established by our hospital pharmacy service for treatment with vismodegib. This may bias the results, as they are not applicable to the general population.

## 2. Materials and Methods

We conducted an observational retrospective study, including histologically confirmed laBCC and/or multiple BCC adult patients, treated with vismodegib from January 2019 to December 2022 at the Department of Dermatology of San Cecilio University Hospital, Granada, Spain. The medical records of patients were reviewed, and the baseline collected data for each patient included age, sex, comorbidities, BCC subtype, primary location, size, self-reported presence of BCC, and previous treatment.

Patients received vismodegib 150 mg once daily until complete resolution of the tumor, disease progression, or unacceptable toxicity. Monthly follow-up was performed, and we registered the clinical evolution and all AEs related to the drug and if these led to treatment interruption or discontinuation. The response status of patients was evaluated after 6 months of treatment. Complete response (CR) was considered as the disappearance of clinical tumor; partial response (PR) was the decrease in tumor volume with persistence; and no response (NR) was when regrowth or no decrease was observed. In addition, we recorded cases in which clinical response was occasionally confirmed by biopsy.

## 3. Results

### 3.1. Study Population

The present study included a total of six patients with biopsy-proven laBCCs and in which other treatments such as surgery or radiotherapy were considered inappropriate due to the patient profile, an expected considerable morbidity and deformity after treatment or the probability of its success. Among all patients, three were females (50%) and three were males (50%), with a mean age of 78.5 (median 80.5, range 63–90) years old at the start of the treatment. Clinical data of patients are summarized in Table 1. Most of our patients (5/6, 83.3%) had some comorbidity at the time of starting treatment. Cardiovascular risk factors such as diabetes mellitus, hypertension or dyslipidemia were present in five patients; cardiovascular disease in the form of coronary heart disease, atrioventricular block or cerebrovascular disease was present in three patients; musculoskeletal disorders such as arthrosis or arthritis rheumatoid were present in two patients; and one patient had a previous medical history of a non-cutaneous solid tumor (diffuse large B cell lymphoma of the parotid gland) in remission at the time of starting the laBCC treatment. The primary location was periocular for three patients (involving the medial canthus in two of them), the nasal area for two patients and multiple primary locations in one case (with a tumor in the abdomen, neck and arm). Mean self-reported tumor presence was 4.8 years (median 2.5, range 2–11 years). The average lesion size based on the longest dimension was 4.75 cm (median 5, range 2.0–8.0 cm). All lesions were biopsy-confirmed BCC with the following histopathological subtypes: nodular (three), nodular and sclerosing (two) or infiltrative sclerosing (one) BCC. Most patients had received no previous treatment, and only two patients had been treated surgically, with affected margins in one of them after multiple surgeries in the nasal pyramid. No patients had metastatic disease.

### 3.2. Efficacy

**Figure 1:**
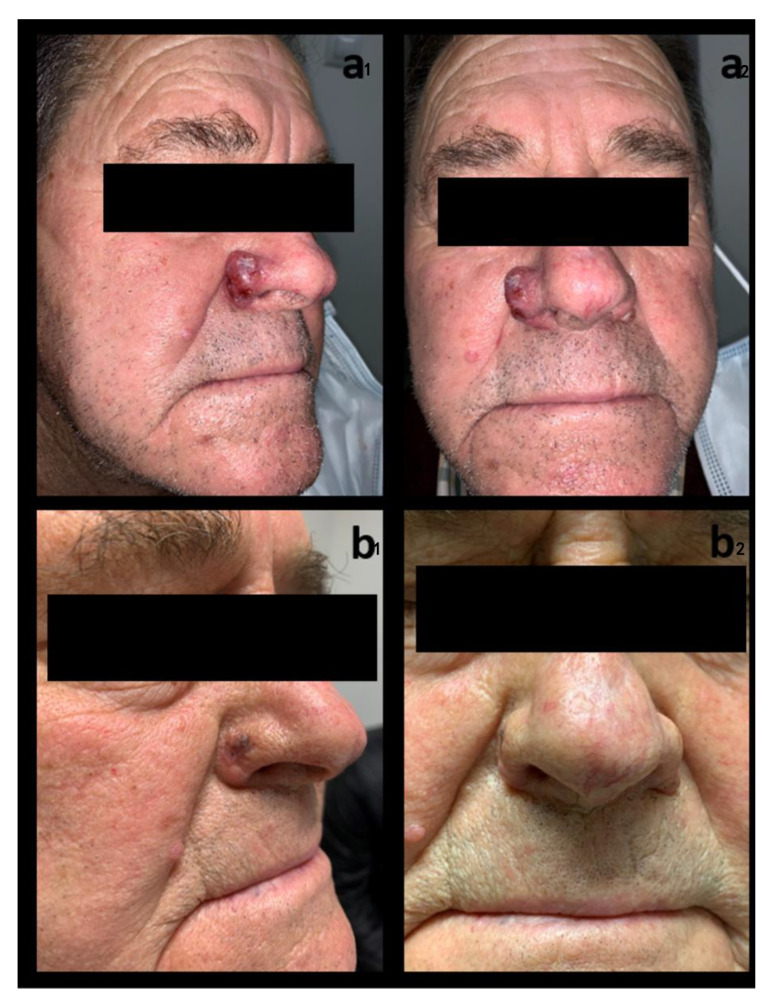
Clinical images of patient 3, (**a1**,**a2**) before starting treatment with vismodegib and (**b1**,**b2**) after completion of treatment (even though based on the clinical images, it is hard to judge whether it is a residual erythematous scar, or the BCC still persists, the histological study conducted after Mohs surgery revealed the absence of neoplasia in all sections studied).

**Figure 2:**
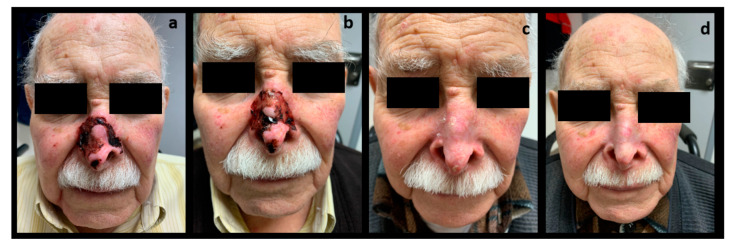
Clinical images of patient 5, (**a**) before starting treatment with vismodegib, (**b**,**c**) during follow-up visits, and (**d**) after completion of treatment.

Table 2 summarizes efficacy and safety outcomes. All patients received 150 mg of oral vismodegib once daily. Mean duration of treatment was 5 months (median 5.5, range 2–6 months). A CR was observed in four cases (66.7%), and a PR was reported in the other two cases (33.3%). Histological confirmation of response was conducted in all patients except one of those who had a PR, in which it was not possible to carry it out due to patient death before the end of therapy. In one of the patients with PR initially (Figure 1), tumor excision surgery using Mohs micrographic surgery following two months with vismodegib treatment was conducted, with reconstruction using dermal fat grafting. However, a histopathological study reported the absence of neoplasia. From the analysis of the drug response time, we obtained a median response onset time of one month (range 1–2 months). On the other hand, the maximum response time was observed at a median of 3.5 months (range 2–6 months). No recurrence was detected, with a mean follow-up duration after treatment discontinuation of 17.8 months (median 20, range 7–26 months). Figure 1 and Figure 2 show the clinical evolution of two patients, one of them with clinical PR and the other with CR.

### 3.3. Adverse Events

In reference to the safety profile, most patients (5/6, 83.3%) had at least one AE, and temporal or permanent dosage adjustment was needed in two patients (33.3%). Dose adjustment consisted of the administration of vismodegib 150 mg on alternate days, which resulted in good tolerability and a reduction in adverse effects. The main adverse effect was muscle spasms, which was present in more than half of our patients (four patients, 66.7%), followed by dysgeusia (two patients, 33.3%), asthenia (two patients, 33.3%) and alopecia (two patients, 33.3%). One patient had increased CK levels at about three times the normal value, leading to a decrease in the vismodegib dose to 150 mg every other day, achieving normalization of the enzyme levels. During follow-up, one patient with PR died five months after starting vismodegib due to other medical reasons (a 90-year-old woman with multiple associated comorbidities). Other AEs such as anorexia, constipation, weight loss, diarrhea or nausea did not occur in our patients.

## 4. Discussion

The management of advanced BCC can be challenging, and standard treatment has not been fully defined [14]. Non-surgical or topical options such as imiquimod, photodynamic therapy or 5-fluorouracil are used in small or superficial lesions but are ineffective in the treatment of advanced tumors. They could be suitable for elderly patients with multiple comorbidities, with the disadvantages of a higher recurrence rates and the absence of histological confirmation of curation [15]. Radiation therapy may be recommended in BCC in patients who are not surgical candidates due to tumor (extent and location) or patient features (comorbidities). In laBCC, radiation has been used with tumor downstaging following surgical resection. This treatment has the advantage of turning tumors that were previously inoperable due to their size into “operable” tumors [16]. However, it has limitations in terms of suboptimal disease control and generates an alteration of the surgical site that will cause complications for subsequent healing. Other disadvantages include dose limitations, the need for multiple visits, and the risk of secondary tumors, and this treatment should not be practiced in previously irradiated fields [9]. Adjuvant chemotherapy for laBCC has not been investigated in randomized controlled trials, and no standard chemotherapeutic regimen exists. Different chemotherapy regimens have been essayed (cisplatin alone or in combination with other agents such as vinblastine, doxorubicin or paclitaxel), with varying degrees of success and durability of responses [17]. However, other therapeutic options and the possibility of inclusion in clinical trials should be strongly preferred.

Vismodegib is an oral medication that received FDA approval in 2012 for the treatment of locally advanced, metastatic or recurrent BCC, in addition to lesions where resection or radiation would cause substantial disfigurement or loss of function and in patients who are not candidates for surgery or radiotherapy [7,18]. Vismodegib has demonstrated clinical efficacy in the treatment of laBCC in two international, multicenter clinical trials, showing clinical response ratios of 68.5% and 60.3% [12,19]. In follow-up studies, the clinical benefit of vismodegib assessed by an independent panel review based on expert clinical judgment was reported in 76% of patients [20]. In our case series, we report a PR or CR in all six patients with laBCC, showing rates higher than the values obtained in these trials. These improved results in terms of efficacy are probably due to our small sample size, less strict clinical criteria used in real-life practice and the lack of multiple biopsies in order to obtain a histologic confirmation of response. In clinical trial studies, the response was assessed using a physical examination and radiology per Response Evaluation Criteria in Solid Tumors (RECIST), with histopathological confirmation of CR in the ERIVANCE-BCC study. In addition, the resolution of laBCC leaves a scar with telangiectasia and irregular vessels, a fibrotic tissue that may resemble tumor-associated fibrosis, making it difficult to differentiate between clinical CR or tumor persistence, so this type of response may have been overestimated among our patients.

Equivalent efficacy in terms of response rates was found in other real-life clinical studies [10,21,22]. In another prospective observational study with a representative sample of United States patients with laBCC (RegiSONIC), the clinical response rate after treatment with vismodegib was 85.1%, with a median duration of response of 17.5 months [23].

In terms of time to response to vismodegib, we found a median time to onset of response of one month and a median time to maximum response of 3.5 months. Our results are somewhat better than those of ERIVANCE, which reported a time to maximum tumor reduction size of 5.5–6.7 months [19].

The initial treatment goal with vismodegib in several of our patients was to achieve sufficient tumor size reduction to allow subsequent conservative surgery. However, in most of our patients, this neoadjuvant treatment became a definitive treatment. Surgery was performed in a single case in which there were clinical data of tumor persistence after two months of treatment with vismodegib. Nevertheless, the final histopathological study confirmed the total remission of the tumor. Several studies have assessed the neoadjuvant use of Hh inhibitors followed by another additional therapy modality such as radiotherapy, achieving a very high CR rate with a low disease-specific progression rate and relatively tolerable toxicity [24,25,26].

In relation with predictive factors of response to vismodegib and the risk of relapse, Juzot et al. [27] showed that increased treatment duration correlates with a decreased risk of relapse, and in the study of Herms et al. [28], laBCC, with a higher risk of relapse, was independently associated with a location on the trunk and limbs. Only one of our patients had an laBCC in one of the high-risk sites for recurrence (trunk), which could explain the fact that there were zero recurrences during the months of follow-up after the end of treatment. Another factor that could explain this fact is the short follow-up time in some of our patients (median 20, range 7–26 months). In another retrospective cohort, the high-risk histologic subtype was a predictor of non-CR [29]; however, this factor did not influence the achievement of CR in our patients.

One of the factors that may contribute to the development of laBCC is diagnostic delay. This problem is present in populations with difficult access to healthcare, or patients with a history of mental illness [30]. The median time of evolution of CCB from the time the patient detected it until it was assessed in our hospital was 4.8 years, which was longer than 10 years in two of our patients, and yet none of them had mental pathology.

The main disadvantage of vismodegib is its high rate of AEs (98% of patients in the STEVIE trial, causing discontinuation of treatment in 31% of patients). The common AEs observed in Hh inhibitors clinical trials are class-related effects and include alopecia, muscle spasm, nausea, dysgeusia, decreased appetite, weight loss and fatigue, with a low percentage of grade 3 or 4 events and discontinuation of treatment [12,19]. Similar adverse effects have been reported in real clinical practice case series [31]. The treatment was generally well tolerated among our patients. Most patients were affected by at least one AE, including fatigue, muscle spasms, dysgeusia and alopecia, which is in accordance with AEs described in the literature. However, AEs were mild (grades 1–2), leading to interruption of treatment in only one of the patients [32].

Table 3 lists AEs reported in our patients and compares them with those reported in the three main clinical trials of vismodegib [12,19,33]. Overall, the most frequent adverse effects, such as muscle spasm, alopecia or dysgeusia, were present in a lower percentage in our patients. However, between 9.3 and 20.1% more of our cases presented with asthenia. Nevertheless, the results are not directly comparable due to the small sample size of our series, together with the differences in treatment duration.

Dysgeusia and weight loss are two common side effects of vismodegib, reported in a high percentage of patients (83% and 54%, respectively) [34]. However, although 33% of patients in our series had dysgeusia, there were no cases of weight loss. Cancer chemotherapy-induced dysgeusia is involved in a reduction in food intake, resulting in weight loss. The short treatment period (two months) could be responsible for the absence of weight loss in one of our patients despite dysgeusia. In the other case, the patient was suffering from cognitive decline and needed to be fed by a caregiver.

Our cohort comprised patients with one or more comorbidities prior to the initiation of treatment, but this did not affect the efficacy or tolerability of treatment. These results are in line with those of another study that showed that vismodegib maintains a high tolerability and good safety in elderly patients with multiple comorbidities, with safety data similar to those reported in long-term follow-up and clinical trials [35].

The temporary interruption of vismodegib is a strategy that allows patients to continue with vismodegib and does not appear to negatively impact the efficacy of these agents [13]. MIKIE study compared two intermittent regimens with 8 week pauses and showed that treatment interruption did not compromise the efficacy of vismodegib, but an intermittent dosing schedule may help mitigate AES since it was associated with a lower number of grade ≥3 AEs compared to the STEVIE trial [33]. In our case, the strategy used was to take vismodegib every other day, achieving good efficacy responses with control of adverse effects, and avoiding treatment interruption for several weeks or permanently. One capsule taken every 48 h to reduce side effects is specified within the label of sonidegib but not in the case of vismodegib [36]. However, to our knowledge, this is the second paper reporting this vismodegib therapeutic scheme with the goal of reducing adverse effects, allowing the continuation of treatment. In a retrospective analysis including 56 patients with laBCC treated with vismodegib, 8 patients received a prophylactic dose reduction (150 mg every other day) from initiation on to avoid severe AEs. The majority of patients reported no AEs (62.5%), two (25%) reported mild AEs, and one (12.5%) reported moderate AEs, with similar efficacies outcomes to patients treated with 150 mg daily [37].

The limitations of our study include its retrospective design and the small sample size. The small size of our sample is due to the fact that, during the study period, few patients met the criteria established by our hospital pharmacy service for treatment with vismodegib. This may lead to biases in the final results because our sample is not representative of the general population. However, it may be similar to the population in regions with patient selection criteria as strict as ours. Therefore, this is a case report whose results in terms of efficacy and safety cannot be extrapolated to the general population. Additional limitations are the nonrandomized treatment assignment and the lack of standardized assessment of efficacy.

## 5. Conclusions

The present study supports the evidence that vismodegib is an effective and safe treatment for laBCC. Its role in unresectable BCC seems to be an important option in these challenging cases, not only as an isolated treatment but also as a neoadjuvant therapy before surgery.

## Figures and Tables

**Table 1:** Patient and tumor characteristics.

	Age	Sex	Comorbidities	Subtype BCC	Tumor Location	Tumor Size (cm)	Self-Reported Presence of BCC (Years)	Previous Treatment
1	63	F	HT	Multiple nodular	Abdomen Neck Arm	8 × 8 5 × 4 6 × 6	10	N
2	86	F	HT, DLP, complete AV block, arthrosis	Nodular and sclerosing	Left inner canthus eye	2 × 2	2	Surgery (relapse)
3	71	M	T2DM, DLP	Nodular	Right nasal wing	2 × 2	1	N
4	90	F	DLP, vascular dementia, DLBCL of the parotid gland, CHD, RA	Nodular and sclerosing	Glabella and right inner canthus eye	3.5 × 4	3	N
5	85	M	HT, T2DM, DLP, CHD	Infiltrative and sclerosing	Wings and nasal dorsum	5 × 4	11	Surgery (with positive margins)
6	76	M	-	Nodular	Right supraciliary and palpebral	6.2 × 2	2	N

Note: Abbreviations: BCC, basal cell carcinoma; M, male; F, female; HT, Hypertension; DLP, dyslipidemia; CHD, coronary heart disease; T2DM, Type 2 diabetes mellitus; DLBC, Diffuse Large B Cell Lymphoma; RA, arthritis rheumatoid; N, no.

**Table 2:** Efficacy and safety outcomes.

Id	Daily Dosage	Time of Treatment (Months)	Clinical Response	Histological Confirmation of Response	Time to Initial/Best Response (Months)	Subsequent Treatment	Follow-Up Post-Treatment	Relapse	Toxicity
1	150 mg	6	CR	Y	1/6	Follow-up	26	N	Alopecia, muscle spasms
2	150 mg–150 mg/48 h	6	CR	Y	1/4	Follow-up	25	N	Asthenia, muscle spasms
3	150 mg	2	CR	Y	1/2	Mohs surgery	20	N	Muscle spasms, dysgeusia
4	150 mg–150 mg/48 h	5	PR	N	1/5	-	-	-	Asthenia, dysgeusia, ↑CK
5	150 mg	6	CR	Y	2/3	Follow-up	11	N	N
6	150 mg	6	CR	Y	1/3	Follow-up	7	N	Alopecia, muscle spasms

Note: ↑CK: Creatin-kinase higher than normal.

**Table 3:** Summary of treatment-emergent adverse events in our cases series in comparison with clinical trials.

	Present Study (n = 6)	Stevie (n = 1215)	Erivance (n = 104)	Mikie		Different Rate
				Group A (n = 114)	Group B (n = 113)	
Any	5 (83.3)	1192 (98.1)	104 (100.0)	107 (93.9)	109 (96.5)	−16.7 to −10.6
Muscle spasm	4 (66.7)	807 (66.4)	74 (71.2)	83 (72.8)	93 (82.3)	−15.6 to +0.3
Alopecia	2 (33.3)	747 (61.5)	69 (66.3)	72 (63.2)	73 (64.6)	−30.0 to −28.2
Dysgeusia	2 (33.3)	663 (54.6)	58 (55.8)	75 (65.8)	75 (66.4)	−33.1 to −21.3
Decreased weight	0	493 (40.6)	54 (51.9)	24 (21.1)	21 (18.6)	
Decreased appetite	0	303 (24.9)	29 (27.9)	21 (18.4)	17 (15.0)	
Asthenia	2 (33.3)	291 (24.0)	0	15 (13.2)	20 (17.7)	+9.3 to +20.1
Nausea	0	218 (17.9)	34 (32.7)	23 (20.2)	15 (13.3)	
Ageusia	0	213 (17.5)	12 (11.5)	14 (12.3)	13 (11.5)	
Fatigue	0	201 (16.5)	45 (43.3)	24 (21.1)	26 (23.0)	
Diarrhea	0	197 (16.2)	28 (26.9)	20 (17.6)	18 (15.9)	
Arthralgia	0	124 (10.2)	17 (16.3)	18 (15.8)	16 (14.2)	
Constipation	0	116 (9.5)	20 (19.2)	0	0	
Vomiting	0	102 (8.4)	18 (17.3)	0	0	
Headache	0	92 (7.6)	15 (14.4)	11 (10)	11 (4)	

## Data Availability

Original data are available upon request to the authors.
